# Supplementary material for: Evaluation of an organisational-level monetary incentive to promote the health and wellbeing of workers in small and medium-sized enterprises: A mixed-methods cluster randomised controlled trial
Source: PLOS Glob Public Health. 2023 Jul 6;3(7):e0001381. doi: 10.1371/journal.pgph.0001381 (PMC10325111; doi:10.1371/journal.pgph.0001381)
Supplement: S1 Checklist — (DOCX) [file pgph.0001381.s001.docx]

| **Section/Topic** | **Item No** | **Checklist item** | **Reported on page No** |
| --- | --- | --- | --- |
| **Title and abstract** | 1a | Identification as a randomised trial in the title | 1 |
|  | 1b | Structured summary of trial design, methods, results, and conclusions (for specific guidance see CONSORT for abstracts) | 2 |
| **Introduction** |  |  |  |
| Background and objectives | 2a | Scientific background and explanation of rationale | 4-5 |
|  | 2b | Specific objectives or hypotheses | 5 |
| **Methods** |  |  |  |
| Trial design | 3a | Description of trial design (such as parallel, factorial) including allocation ratio | 5, 7 |
|  | 3b | Important changes to methods after trial commencement (such as eligibility criteria), with reasons | 5-6 |
| Participants | 4a | Eligibility criteria for participants | 6-7 |
|  | 4b | Settings and locations where the data were collected | 5-6 and published protocol (referenced in manuscript, 15) |
| Interventions | 5 | The interventions for each group with sufficient details to allow replication, including how and when they were actually administered | 8-11 |
| Outcomes | 6a | Completely defined pre-specified primary and secondary outcome measures, including how and when they were assessed | 11-13. supplementary figure 1, Supplementary Table S3 |
|  | 6b | Any changes to trial outcomes after the trial commenced, with reason | NA |
| Sample size | 7a | How sample size was determined | 13 |
|  | 7b | When applicable, explanation of any interim analyses and stopping guidelines | NA |
| Randomisation: |  |  |  |
| Sequence generation | 8a | Method used to generate the random allocation sequence | 7 |
|  | 8b | Type of randomisation; details of any restriction (such as blocking and block size) | 8, 13 and reference to the trial protocol |
| Allocation concealment mechanism | 9 | Mechanism used to implement the random allocation sequence (such as sequentially numbered containers), describing any steps taken to conceal the sequence until interventions were assigned | In published protocol (15) |
| Implementation | 10 | Who generated the random allocation sequence, who enrolled participants, and who assigned participants to interventions | Page 8 and in published protocol (15) |
| Blinding | 11a | If done, who was blinded after assignment to interventions (for example, participants, care providers, those assessing outcomes) and how | Page 8 and in published protocol (15) |
|  | 11b | If relevant, description of the similarity of interventions | 9, 11 |
| Statistical methods | 12a | Statistical methods used to compare groups for primary and secondary outcomes | 13-14 |
|  | 12b | Methods for additional analyses, such as subgroup analyses and adjusted analyses | NA |
| **Results** |  |  |  |
| Participant flow (a diagram is strongly recommended) | 13a | For each group, the numbers of participants who were randomly assigned, received intended treatment, and were analysed for the primary outcome | Figure S1 |
|  | 13b | For each group, losses and exclusions after randomisation, together with reasons | Figure S1 |
| Recruitment | 14a | Dates defining the periods of recruitment and follow-up | Figure S1 |
|  | 14b | Why the trial ended or was stopped | NA |
| Baseline data | 15 | A table showing baseline demographic and clinical characteristics for each group | 15, 16 (table 2), Supplementary Table S5 |
| Numbers analysed | 16 | For each group, number of participants (denominator) included in each analysis and whether the analysis was by original assigned groups | Supplementary Table S5 |
| Outcomes and estimation | 17a | For each primary and secondary outcome, results for each group, and the estimated effect size and its precision (such as 95% confidence interval) | Text: 16-18 ,20-23, Table 3, Supplementary Table S4, Supplementary Table S6, Supplementary Table S8, |
|  | 17b | For binary outcomes, presentation of both absolute and relative effect sizes is recommended | Supplementary Table S8 |
| Ancillary analyses | 18 | Results of any other analyses performed, including subgroup analyses and adjusted analyses, distinguishing pre-specified from exploratory |  |
| Harms | 19 | All important harms or unintended effects in each group (for specific guidance see CONSORT for harms) | NA |
| **Discussion** |  |  |  |
| Limitations | 20 | Trial limitations, addressing sources of potential bias, imprecision, and, if relevant, multiplicity of analyses | 26 |
| Generalisability | 21 | Generalisability (external validity, applicability) of the trial findings | 26 |
| Interpretation | 22 | Interpretation consistent with results, balancing benefits and harms, and considering other relevant evidence | 23-28 |
| **Other information** |  |  |  |
| Registration | 23 | Registration number and name of trial registry | 3, 14 |
| Protocol | 24 | Where the full trial protocol can be accessed, if available | Reference 15 and uploaded in supporting information |
| Funding | 25 | Sources of funding and other support (such as supply of drugs), role of funders | 4, 9, 14, 35 |
