# Supplementary material for: Evaluation of an organisational-level monetary incentive to promote the health and wellbeing of workers in small and medium-sized enterprises: A mixed-methods cluster randomised controlled trial
Source: PLOS Glob Public Health. 2023 Jul 6;3(7):e0001381. doi: 10.1371/journal.pgph.0001381 (PMC10325111; doi:10.1371/journal.pgph.0001381)
Supplement: S1 Text — Table A. Self-assessment criteria description. Table B. Self-assessment criteria payments. Fig A. Effect of Incentive on Employer “Offer”–A Simplified Causal Chain Linking Intervention to Outcomes. Table C. Quantitative outcome measures. Table D. Process evaluation codes. Table E. Positive action–employees’ perception of amount of action. Table F. Summary of employee-level covariates at baseline and endline (11 months) by group. Fig B. Employee Perception of Employer and Employee Behaviour Change Outcomes. Table G. Employee Outcomes Model-Based Analyses. Table H. Summary of. crude employee-level outcomes by baseline/end line and trial group (%). Table I. Model-based estimates of the reactivity effect and endline versus baseline. Table J. Summary of employer-level crude outcomes by baseline/endline (%). (DOCX) [file pgph.0001381.s002.docx]

# Supplementary Text S1: Network meetings

Network meetings were separate for each trial group (Group 1; Group 2; and Group 3). Each meeting followed the same format. It included a 1) presentation to increase health awareness (for instance presentation from Prostate Cancer UK), 2) group discussions and 3) visual presentations on how to complete the self-assessment criteria (Supplementary Tables S1-S2). Network meetings were facilitated by trained members of the West Midland Combined Authority (local government group) and qualitatively observed by trained researchers. Participants discussed challenges they had experienced so far (or anticipated) as part of implementing a health and wellbeing offer. Participants were invited to join walking meetings and discuss victories in implementing health and wellbeing offers. Some attendees asked questions about the type of evidence which could be uploaded for specific sections of the criteria in the online dashboard (see Supplementary Tables S1-S2).

# Table A in S1 Text: Self-assessment criteria description

| **Domain** | **Description** | **Requirements** |
| --- | --- | --- |
| **Dashboard 1** | Organisation login details to access the form | <https://www.wmca.org.uk/what-we-do/thrive/thrive-at-work/app/#!/login/> |
| **Dashboard 2** | Health need assessment | Organisation to complete a health need assessment to identify key wellbeing priorities and to assign a health and wellbeing lead |
| **Dashboard 3** | Mental health | Organisation to upload evidence of mental health initiatives and progress throughout the trial |
| **Dashboard 4** | MSK health | Organisation to upload evidence of MSK health initiatives and progress throughout the trial |
| **Dashboard 5** | Lifestyle health | Organisation to upload evidence of lifestyle health initiatives and progress throughout the trial |

# Table B in S1 Text: Self-assessment criteria payments

| **Theme** | **Number of criteria** | **Time/Resource requirements** | **Senior Level And/ Or Financial Input requirements** | **Percentage of final grant per theme** |
| --- | --- | --- | --- | --- |
| **Enablers of health** | 22 | High | +++ | 44% |
| **Mental health** | 5 | Medium/High | ++ | 25% |
| **Musculoskeletal health** | 2 | Low/Medium | + | 10% |
| **Lifestyle health** | 14 | Medium | ++ | 21% |

**Note.** There was a maximum of 43 criteria that each SME could achieve across each of four themes, which covered 22 criteria for enablers of health, 5 for mental health, 2 for musculoskeletal health, and 14 for lifestyle health. Payments differed depending on the time and resources required to achieve the criteria. Key organisational enablers of health were viewed to facilitate the conditions needed to improve mental health, musculoskeletal health, and lifestyle health, and required a high level of senior management input to achieve (examples included attendance management and organisational policies and procedures). The payment formula was thus as follows:

Size of payment = Trial group payment size * Number of staff * [0.44*(number of enablers of health criteria achieved) * 0.25(number of mental health criteria achieved) * 0.10(number of musculoskeletal health criteria achieved) * 0.21(number of lifestyle health criteria achieved)]

**Fig A in S1 Text: Effect of Incentive on Employer “Offer” – A Simplified Causal Chain Linking Intervention to Outcomes**

**
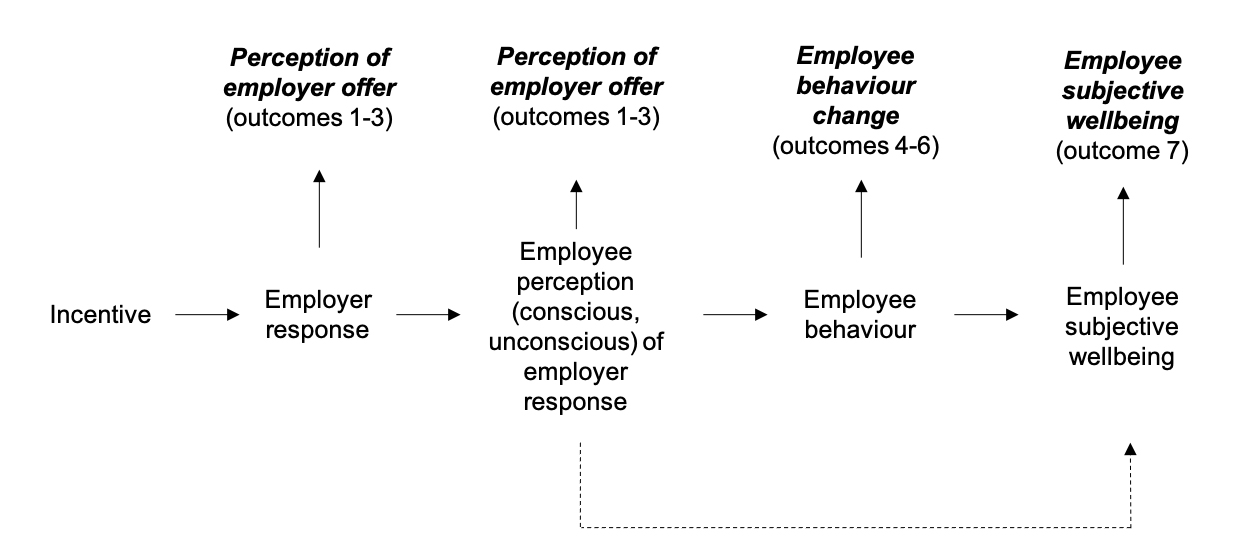
**

*Note*. Change may occur without conscious perception, consistent with psychological literature on unconscious influences on behaviour [Bargh JA. Our unconscious mind. Sci Am. 2014;310(1):30-7. DOI: 10.1038/scientificamerican0114-30].

**Table C in S1 Text: Quantitative Outcome Measures**

| **Outcome** | | **Question Asked** | **Asked of Employer/Employee** |
| --- | --- | --- | --- |
| Perception of employer offer | 1.Positive action (**primary outcome)** | Does your organisation take positive action on health and wellbeing? ^a^ | Both |
|  | 2.Awareness of information | In the last six months, has your organisation provided information about any of the following? ^b^ | Both |
|  | 3.Awareness of activities/services | In the last six months, has your organisation provided activities or services for any of the following? ^b^ | Both |
| Employee behaviour change | 4.Conscious effort | In the last six months, have you made a conscious effort to improve your…? ^b^ | Employee |
|  | 5.Taking part at work | In previous questions, you said that your organisation provided information, activities, and/or services related to any of the following. ^b^ Did you use or take part in the initiative(s)?  In previous questions, you said that your organisation provided information, activities, and/or services related to any of the following. ^b^ Did your employees use or take part in the initiative(s)? ^c^ | Both ^c^ |
|  | 6. Taking part outside work | In the last six months, have you joined a group organisation or used a service that is outside your workplace related to your…? ^b^ | Employee |
| Subjective wellbeing | 7. Satisfaction, worthwhile, happy, anxious  Response: 0, not at all to 10, completely | Overall, how satisfied are you with your life nowadays?  Overall, to what extent do you feel the things you do in your life are worthwhile?  Overall, how happy did you feel yesterday?  Overall, how anxious did you feel yesterday? | Employee |

*Note*. All outcomes are binary, dichotomous, yes/no - unless stated otherwise.

^a^ If participants answered ‘yes’ they were asked ‘how much action’ (very little, some, quite a bit, a lot) - results presented in Supplementary Table S4 and produce similar results to the positive action question;

^b^ Mental health; MSK health; Healthy lifestyles (e.g. smoking, alcohol use, diet, physical activity, active travel?);

^c^ Questions presented separately because the wording of this question (number 5) differed for employee and employer.

**Table D in S1 Text:** **Process Evaluation Codes**

| **Code** | **Sub-code** | **WMCA** | **Employers** | **Employees** |
| --- | --- | --- | --- | --- |
| **Background information** |  | X | X | X |
| **Health and wellbeing for staff** | Interest and engagement |  |  | X |
|  | Ambitions, goals and plans |  |  | X |
|  | Activities |  |  | X |
|  | Barriers and enablers |  |  | X |
|  | Control and empowerment |  |  | X |
| **Sign up to trial** | Motivation |  | X |  |
|  | Ambition and goals |  | X |  |
|  | Knowledge and awareness |  | x |  |
|  | Barriers and enablers |  | X |  |
| **WMCA communications** | Volume and type | X | X |  |
|  | Awareness, engagement and interest | X | X |  |
|  | Understanding | X | X |  |
|  | Problems and issues | X | X |  |
|  | Impact | X | X |  |
|  | Areas for improvement | X | X |  |
| **Employer communications** | Volume and type |  | X | X |
|  | Awareness, engagement and interest |  | X | X |
|  | Effectiveness |  |  | X |
|  | Understanding |  | X | X |
|  | Problems and issues |  | X | X |
|  | Impact |  | X | X |
|  | Areas for improvement | X | X |  |
| **Needs assessment** | WMCA guidance | X | X |  |
|  | Whether conducted |  | X |  |
|  | Staff consultation |  | X | X |
|  | Learnings |  | X |  |
|  | Problems and issues |  | X |  |
|  | Barriers and enablers |  | X |  |
|  | Impact |  | X |  |
|  | Areas for improvement | X | X |  |
| **Previous health and wellbeing offer** | Offer |  | X | X |
|  | Beneficiaries |  | X |  |
|  | Awareness, engagement and interest |  | X | X |
|  | Understanding |  | X | X |
|  | Participation |  | X | X |
|  | Barriers and enablers |  | X | X |
|  | Self-assessment | X | X |  |
|  | Problems and issues |  | X |  |
|  | Impact |  | X | X |
|  | Areas for improvement/feedback |  | X | X |
| **New health and wellbeing offer** | Changes planned |  | X |  |
|  | Changes implemented |  | X | X |
|  | Beneficiaries |  | X |  |
|  | Awareness, engagement and interest |  | X | X |
|  | Understanding |  | X | X |
|  | Participation |  | X | X |
|  | Barriers and enablers |  | X | X |
|  | Problems and issues |  | X |  |
|  | Prioritisation and decision-making |  | x |  |
|  | Impact |  | X | X |
|  | Areas for improvement/feedback |  | X | X |
| **Grant** | Conditions and requirements | X | X |  |
|  | Understanding | X | X |  |
|  | Decision-making |  | X |  |
|  | Spending | X | X |  |
|  | Other resources |  | X |  |
|  | Barriers and enablers | X | X |  |
|  | Problems and issues | X | X |  |
|  | Impact | X | X |  |
|  | Areas for improvement | X | X |  |
| **Commitment** | Guidance | X | X |  |
|  | Conditions and requirements | X | X |  |
|  | Awareness, engagement and interest | X | X | X |
|  | Understanding | X | X | X |
|  | Use and implementation | X | X |  |
|  | Barriers and enablers | X | X |  |
|  | Problems and issues | X | X |  |
|  | Impact | X | X |  |
|  | Areas for improvement | X | X |  |
| **Toolkit** | Guidance | X | X |  |
|  | Awareness engagement and interest | X | X | X |
|  | Understanding | X | X | X |
|  | Use | X | X | X |
|  | Barriers and enablers | X | X |  |
|  | Problems and issues | X | X |  |
|  | Impact | X | X |  |
|  | Areas for improvement | X | X |  |
| **Network meetings** | Format and content | X | X |  |
|  | Awareness, engagement and interest | X | X |  |
|  | Understanding | X | X |  |
|  | Participation | X | X |  |
|  | Barriers and enablers | X | X |  |
|  | Problems and issues | X | X |  |
|  | Impact | X | X |  |
|  | Areas for improvement | X | X |  |

# Table E in S1 Text: Positive action – employees’ perception of amount of action.

| **Outcome** | | **High versus no incentive** | | **Low versus no incentive** | | **Reactivity effect** | | **Endline versus baseline** | |
| --- | --- | --- | --- | --- | --- | --- | --- | --- | --- |
| **Perception of employer offerings** |  | RR (95% CrI) | Prob. | RR (95% CrI) | Prob. | RR (95% CrI) | Prob. | RR (95% CrI) | Prob. |
|  | My organisation takes positive action ('quite a bit' or 'a lot' vs. ‘no’ or ‘some’ positive action) | 1.06  (0.80, 1.39) | 71% | 0.92  (0.59, 1.17) | 26% | 0.94  (0.61, 1.22) | 30% | **1.25**  **(1.02, 1.78)** | **99%** |

Note. Results for secondary positive action question as relative risks (RR), and the probability the intervention effect is positive (prob.:RR>1 or absolute effect > 0). Results with **“strong evidence” (probability >95%)**, “fair evidence” (probability 80% - 95%) of an effect are highlighted, and “little evidence” (probability <80%) are not highlighted.

If employees responded that ‘yes’, their organisation takes positive action on health and wellbeing (primary outcome), they were subsequently asked ‘how much action does it take?’ (very little, some quite a bit, or a lot of action). Employees responding ‘quite a bit’ or ‘a lot’ were compared to those who responded ‘some or ‘no’.

# Table F in S1 Text: Summary of employee-level covariates at baseline and endline (11 months) by group

All values are % unless otherwise stated

|  | **Group 1** | **Group 2** | **Group 3** | **Group 4** |
| --- | --- | --- | --- | --- |
| **Baseline** | | | | |
| n | 279 | 215 | 226 | N/A |
| Age, mean (sd) | 38.1 (12.4) | 37.5 (11.7) | 35.8 (12.1) | N/A |
| Female | 49 | 49 | 48 | N/A |
| Education (level 1, 1-4 GCSEs C or above) | 6 | 4 | 4 | N/A |
| Education (level 2, 5+ GCSEs C or above) | 13 | 9 | 11 | N/A |
| Education (level 3, 2+ A-levels) | 20 | 20 | 32 | N/A |
| Education (level 4, bachelors degree and higher) | 54 | 63 | 46 | N/A |
| Ethnicity (Black/African/Caribbean/Black British) | 5 | 5 | 4 | N/A |
| Ethnicity (Asian/Asian British) | 10 | 10 | 6 | N/A |
| Ethnicity (White British & White other) | 82 | 83 | 86 | N/A |
| **Endline** | | | | |
| n | 309 | 218 | 145 | 195 |
| Age, mean (sd) | 38.5 (12.7) | 38.8 (12.2) | 37.5 (11.4) | 38.5 (13.2) |
| Female | 50 | 50 | 49 | 50 |
| Education (level 1, 1-4 GCSEs C or above) | 5 | 3 | 2 | 3 |
| Education (level 2, 5+ GCSEs C or above) | 14 | 10 | 8 | 10 |
| Education (level 3, 2+ A-levels) | 23 | 26 | 21 | 24 |
| Education (level 4, bachelors degree and higher) | 55 | 56 | 60 | 59 |
| Ethnicity (Black/African/Caribbean/Black British) | 5 | 5 | 2 | 4 |
| Ethnicity (Asian/Asian British) | 10 | 11 | 8 | 8 |
| Ethnicity (White British & White other) | 83 | 81 | 87 | 86 |

**Fig B in S1 Text: Employee Perception of Employer and Employee Behaviour Change Outcomes**

**
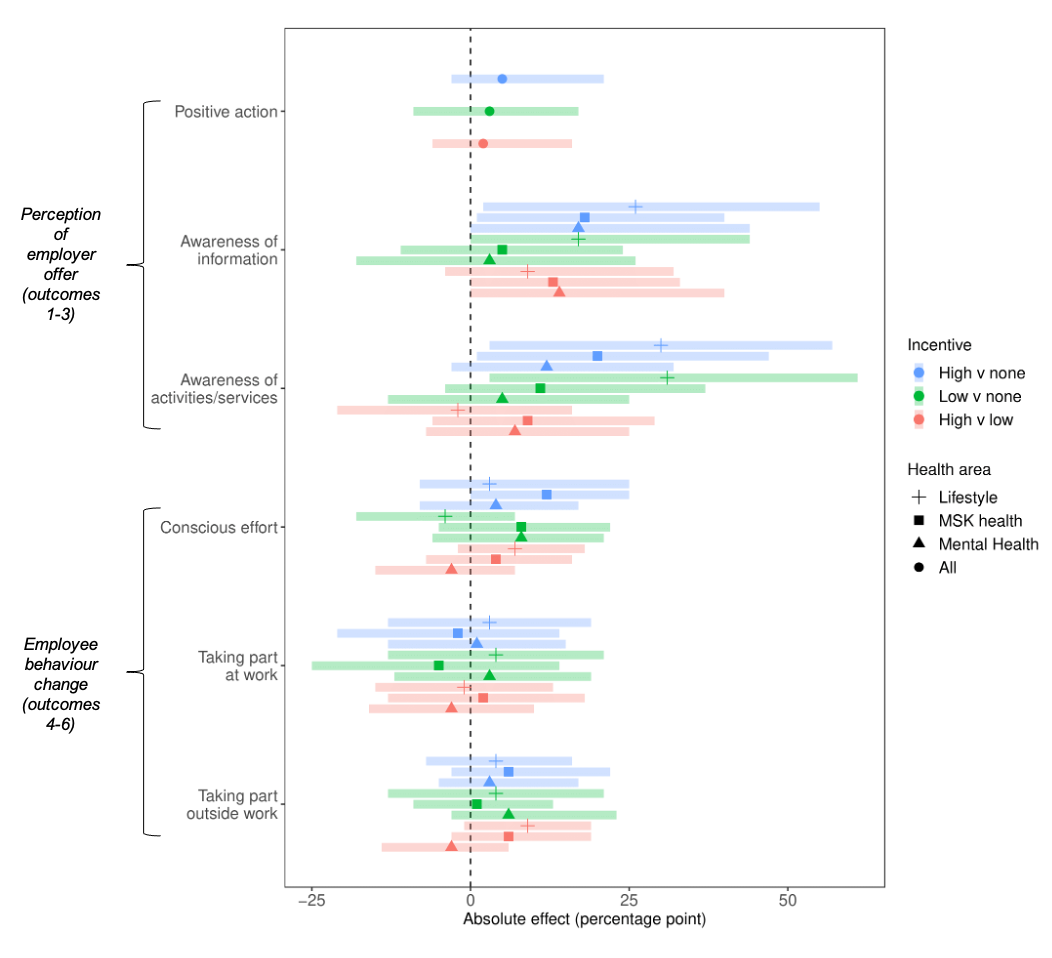
**

*Note*. Absolute intervention effects (percentage point) and 95% credible intervals. Obtained from model-based analyses comparing high versus no incentive, low versus no incentive, and high versus low incentive conditions.

**Table G in S1 Text:** **Employee Outcomes Model-Based Analyses**

| **Outcome** |  | **High versus no incentive** | | | **Low versus no incentive** | | | **High versus low incentive** | | |
| --- | --- | --- | --- | --- | --- | --- | --- | --- | --- | --- |
| Question | Category | RR (95% CrI) | Abs. eff. (pp; 95% CrI) | Prob. | RR (95% CrI) | Abs. eff. (pp; 95% CrI) | Prob. | RR (95% CrI) | Abs. eff. (pp; 95% CrI) | Prob. |
| Positive action (1) | | 1.06  (0.97, 1.35) | 5  (-3, 21) | 91% | 1.04  (0.89, 1.27) | 3  (-9, 17) | 77% | 1.06  (0.97, 1.35) | 2  (-6, 16) | 73% |
| Awareness of information (2) | Mental health | **1.60**  **(1.00, 3.93)** | **17**  **(0, 44)** | **98%** | 1.11  (0.53, 2.11) | 3  (-18, 26) | 64% | **1.49**  **(1.00, 3.63)** | **14**  **(0, 40)** | **97%** |
|  | MSK | **2.03**  **(1.05, 4.25)** | **18**  **(1, 40)** | **99%** | 1.25  (0.58, 2.41) | 5  (-11, 24) | 72% | **1.70**  **(1.00, 3.50)** | **13**  **(0, 33)** | **97%** |
|  | Lifestyle | **2.20**  **(1.02, 6.64)** | **26**  **(2, 55)** | **>99%** | **1.71**  **(1.00, 4.26)** | **17**  **(0, 44)** | **98%** | 1.26  (0.93, 2.54) | 9  (-4, 32) | 90% |
| Awareness of activity/service (3) | Mental health | 1.48  (0.90, 2.91) | 12  (-3, 32) | 94% | 1.21  (0.62, 2.28) | 5  (-13, 25) | 72% | 1.27  (0.81, 2.34) | 7  (-7, 25) | 83% |
|  | MSK | **2.62**  **(1.11, 6.35)** | **20**  **(1, 47)** | **99%** | 1.84  (0.74, 4.29) | 11  (-4, 37) | 90% | 1.51  (0.79, 3.23) | 9  (-6, 29) | 88% |
|  | Lifestyle | **2.86**  **(1.05, 8.62)** | **30**  **(3, 58)** | **>99%** | **3.03**  **(1.05, 9.74)** | **31**  **(3, 61)** | **>99%** | 0.99  (0.57, 1.54) | -2  (-21, 16) | 40% |

| Conscious effort (4) | Mental health | 1.08  (0.85, 1.39) | 4  (-8, 17) | 77% | 1.14  (0.90, 1.51) | 8  (-6, 21) | 88% | 0.95  (0.75, 1.15) | -3  (-15, 7) | 27% |
| --- | --- | --- | --- | --- | --- | --- | --- | --- | --- | --- |
|  | MSK | **1.32**  **(1.00, 1.81)** | **12**  **(-0, 25)** | **97%** | 1.22  (0.88, 1.67) | 8  (-5, 22) | 90% | 1.10  (0.86, 1.44) | 4  (-7, 16) | 76% |
|  | Lifestyle | 1.04  (0.89, 1.18) | 3  (-8, 12) | 76% | 0.95  (0.75, 1.10) | -4  (-18, 7) | 29% | 1.10  (0.97, 1.33) | 7  (-2, 18) | 92% |
| Taking part at work (5) | Mental health | 1.02  (0.68, 1.43) | 1  (-13, 15) | 55% | 1.08  (0.71, 1.57) | 3  (-12, 19) | 67% | 0.96  (0.66, 1.30) | -3  (-16, 10) | 34% |
|  | MSK | 0.96  (0.59, 1.31) | -2  (-21, 14) | 41% | 0.92  (0.53, 1.28) | -5  (-25, 14) | 33% | 1.06  (0.76, 1.55) | 2  (-13, 18) | 61% |
|  | Lifestyle | 1.07  (0.74, 1.52) | 3  (-13, 19) | 68% | 1.08  (0.73, 1.57) | 4  (-13, 21) | 69% | 1.00  (0.73, 1.34) | -1  (-15, 13) | 47% |
| Taking part outside work (6) | Mental health | 1.28  (0.64, 2.25) | 3  (-5, 17) | 74% | 1.54  (0.76, 2.78) | 6  (-3, 23) | 87% | 0.87  (0.49, 1.42) | -3  (-14, 6) | 25% |
|  | MSK | 1.44  (0.78, 2.41) | 6  (-3, 22) | 88% | 1.05  (0.53, 1.81) | 1  (-9, 13) | 50% | 1.43  (0.86, 2.40) | 6  (-3, 19) | 90% |
|  | Lifestyle | 1.13  (0.80, 1.50) | 4  (-7, 16) | 77% | 0.87  (0.56, 1.21) | 4  (-13, 21) | 21% | **1.33**  **(0.97, 1.84)** | **9**  **(-1, 19)** | **96%** |

*Note.* Analyses reporting relative risks (RR), absolute risk differences, and the probability the intervention effect is positive (prob.:RR>1 or absolute effect > 0). Results with “strong evidence” (probability >95%), “fair evidence” (probability 80% - 95%) of an effect are highlighted, and “little evidence” (probability <80%) are not highlighted.

# Table H in S1 Text: Summary of crude employee-level outcomes by baseline/end line and trial group (%)

|  |  | **Group 1** | | **Group 2** | | **Group 3** | | **Group 4** |
| --- | --- | --- | --- | --- | --- | --- | --- | --- |
|  |  | **Baseline** | **Endline** | **Baseline** | **Endline** | **Baseline** | **Endline** | **Endline** |
| Positive action (1) | | 84 | 96 | 82 | 94 | 87 | 92 | 92 |
| Awareness of information (2) | Mental health | 38 | 87 | 45 | 76 | 50 | 80 | 71 |
|  | MSK | 22 | 59 | 16 | 44 | 20 | 41 | 35 |
|  | Lifestyle | 34 | 83 | 34 | 72 | 44 | 59 | 65 |
| Awareness of activity/ service (3) | Mental health | 29 | 66 | 26 | 57 | 34 | 60 | 53 |
|  | MSK | 16 | 43 | 10 | 31 | 15 | 21 | 28 |
|  | Lifestyle | 28 | 68 | 27 | 65 | 31 | 38 | 46 |
| Conscious effort to improve (4) | Mental health | 51 | 62 | 57 | 66 | 61 | 59 | 63 |
|  | MSK | 36 | 57 | 39 | 54 | 38 | 45 | 47 |
|  | Lifestyle | 76 | 85 | 80 | 80 | 79 | 83 | 77 |
| Taking part at work (5) | Mental health | 48 | 49 | 51 | 52 | 51 | 50 | 49 |
|  | MSK | 73 | 61 | 69 | 58 | 59 | 63 | 62 |
|  | Lifestyle | 54 | 70 | 52 | 70 | 58 | 68 | 65 |
| Taking part outside of work (6) | Mental health | 8 | 14 | 15 | 18 | 14 | 12 | 14 |
|  | MSK | 13 | 26 | 16 | 21 | 15 | 20 | 19 |
|  | Lifestyle | 33 | 39 | 43 | 30 | 31 | 35 | 33 |

# Table I in S1 Text: Model-based estimates of the reactivity effect and endline versus baseline

| **Outcome** |  | **Reactivity effect** | | | | **Endline versus baseline** | | |
| --- | --- | --- | --- | --- | --- | --- | --- | --- |
| Question | Category | RR (95% CrI) | Abs. eff. (pp; 95% CrI) | Prob. | RR (95% CrI) | | Abs. eff. (pp; 95% CrI) | Prob. |
| Positive action (1) | | 1.00  (0.78, 1.18) | 0  (-16, 12) | 50% | **1.11**  **(1.00, 1.47)** | | **7**  **(0, 24)** | **98%** |
| Awareness of information (2) | Mental health | 1.15  (0.57, 2.23) | 4  (-17, 28) | 67% | **2.17**  **(1.04, 5.44)** | | **26**  **(3, 49)** | **>99%** |
|  | MSK | 1.04  (0.46, 1.99) | 0  (-17, 18) | 48% | **2.60**  **(1.22, 5.22)** | | **20**  **(2, 41)** | **>99%** |
|  | Lifestyle | 0.78  (0.27, 1.34) | -9  (-33, 10) | 16% | **2.53**  **(1.06, 6.50)** | | **27**  **(4, 51)** | **>99%** |
| Awareness of activity/service (3) | Mental health | 1.04  (0.49, 1.90) | 0  (-19, 20) | 50% | **2.37**  **(1.14, 5.00)** | | **24**  **(4, 44)** | **>99%** |
|  | MSK | 0.65  (0.20, 1.41) | -7  (-31, 6) | 13% | **3.00**  **(1.32, 6.41)** | | **17**  **(1, 42)** | **>99%** |
|  | Lifestyle | 0.65  (0.18, 1.24) | -12  (-38, 5) | 8% | **2.42**  **(1.07, 5.67)** | | **20**  **(1, 44)** | **>99%** |
| Conscious effort (4) | Mental health | 0.91  (0.63, 1.18) | -5  (-19, 9) | 24% | 1.14  (0.94, 1.45) | | 7  (-3, 17) | 91% |
|  | MSK | 0.92  (0.61, 1.25) | -4  (-16, 9) | 30% | **1.32**  **(1.03, 1.75)** | | **11**  **(1, 21)** | **99%** |
|  | Lifestyle | 1.08  (0.95, 1.25) | 6  (-4, 16) | 89% | 0.98  (0.88, 1.08) | | -1  (-9, 6) | 38% |
| Taking part at work (5) | Mental health | 1.02  (0.64, 1.52) | 0  (-16, 17) | 50% | 1.00  (0.70, 1.37) | | 0  (-13, 13) | 48% |
|  | MSK | 1.03  (0.66, 1.51) | 1  (-19, 21) | 55% | 0.87  (0.56, 1.11) | | -8  (-25, 6) | 14% |
|  | Lifestyle | 1.06  (0.70, 1.57) | 3  (-15, 21) | 63% | 1.19  (0.90, 1.72) | | 8  (-5, 23) | 89% |
| Taking part outside work (6) | Mental health | 0.94  (0.42, 1.77) | -1  (-11, 9) | 36% | 1.26  (0.70, 2.02) | | 3  (-4, 12) | 78% |
|  | MSK | 1.18  (0.59, 2.07) | 2  (-8, 15) | 66% | 1.28  (0.74, 2.04) | | 4  (-4, 14) | 81% |
|  | Lifestyle | 1.07  (0.72, 1.47) | 2  (-10, 14) | 63% | 0.94  (0.70, 1.21) | | -2  (-11, 7) | 30% |

*Note*. Results for dichotomous outcomes as relative risks (RR), absolute risk differences, and the probability the intervention effect is positive (prob.:RR>1 or absolute effect > 0). Results with **“strong evidence” (probability >95%)**, “fair evidence” (probability 80% - 95%) of an effect are highlighted, and “little evidence” (probability <80%) are not highlighted.

| Outcome | **Reactivity effect** | | **Endline versus baseline** | |
| --- | --- | --- | --- | --- |
|  | Abs. eff. (95% CrI) | Prob. | Abs. eff. (95% CrI) | Prob. |
| Life satisfaction (0-10) | -0.21  (-0.64, 0.22) | 17% | **0.44**  **(0.14, 0.75)** | **>99%** |
| Worthwhile (0-10) | -0.09  (-0.59, 0.29) | 34% | 0.20  (-0.09, 0.49) | 91% |
| Happiness (0-10) | -0.20  (-0.71, 0.31) | 22% | **0.42**  **(0.05, 0.78)** | **99%** |
| Anxiety (0-10) | -0.02  (-0.69, 0.66) | 48% | -0.28  (-0.80, 0.23) | 12% |

*Note*. Results for continuous wellbeing outcomes as absolute effects and 95% credible intervals along with the probability (prob.) the effect is greater than zero. Results with “strong evidence” (probability >95%), “fair evidence” (probability 80% - 95%) of an effect are highlighted, and “little evidence” (probability <80%) are not highlighted.

# Table J in S1 Text: Summary of employer-level crude outcomes by baseline/endline (%)

|  |  | **Group 1** | | **Group 2** | | **Group 3** | | **Group 4** |
| --- | --- | --- | --- | --- | --- | --- | --- | --- |
|  |  | **Baseline** | **Endline** | **Baseline** | **Endline** | **Baseline** | **Endline** | **Endline** |
| Positive action (1) | | 87 | 100 | 85 | 95 | 96 | 100 | 100 |
| Awareness of information (2) | Mental health | 48 | 100 | 52 | 91 | 77 | 88 | 86 |
|  | MSK | 19 | 66 | 26 | 68 | 38 | 56 | 57 |
|  | Lifestyle | 42 | 100 | 22 | 91 | 50 | 81 | 67 |
| Awareness of activity/ service (3) | Mental health | 48 | 100 | 52 | 91 | 77 | 88 | 86 |
|  | MSK | 19 | 66 | 26 | 68 | 38 | 56 | 57 |
|  | Lifestyle | 42 | 100 | 22 | 91 | 50 | 81 | 67 |
| Taking part at work (5) | Mental health | 52 | 86 | 48 | 77 | 69 | 69 | 67 |
|  | MSK | 29 | 41 | 36 | 50 | 35 | 63 | 48 |
|  | Lifestyle45 | 45 | 93 | 32 | 86 | 50 | 8 | 67 |

**Employer sampling**

One employer per SME volunteered to be surveyed.

**Employer perception of offering**

*Positive action*

A greater proportion of employers perceived that they had taken “positive action” in the high incentive group (mean change 13pp) in comparison to the low incentive group (10pp), and the control group 3 (4pp).

*Awareness of information*

Likewise, employers for both the high and low incentives perceived themselves to have increased their provision of information concerning mental, MSK, and lifestyle health. Generally speaking, employers in the higher dose of the incentive presented a larger mean change for information provision across all three health conditions in comparison to other trial groups. For instance, the high incentive group reported higher change (52pp) in mental health information provision in comparison to the low incentive (39pp), and the control group 3 (11pp).

*Awareness of activities/services*

Employers in the high incentive group perceived themselves to have increased their provision of activities/services across all three health conditions in comparison to other trial groups.

**Employer perception of employee behaviour change**

In contrast to the low impact reported by employees, employers in both high and low incentive groups perceived a higher employee engagement in workplace initiatives for mental health and lifestyle relative to the control (Group3). For instance, both high (48pp) and low (54pp) incentive groups perceived higher employee engagement in life-style initiatives in comparison to the control group 3 (31pp).
