# Supplementary material for: Evaluation of an organisational-level monetary incentive to promote the health and wellbeing of workers in small and medium-sized enterprises: A mixed-methods cluster randomised controlled trial
Source: PLOS Glob Public Health. 2023 Jul 6;3(7):e0001381. doi: 10.1371/journal.pgph.0001381 (PMC10325111; doi:10.1371/journal.pgph.0001381)
Supplement: S1 File — (PDF) [file pgph.0001381.s003.pdf]

Thrive at Work Wellbeing Programme Collaboration *BMC Public Health* (2019) 19:493
https://doi.org/10.1186/s12889-019-6582-y

BMC Public Health

STUDY PROTOCOL

Open Access

Evaluation of a policy intervention to promote the health and wellbeing of workers in small and medium sized enterprises – a cluster randomised controlled trial

Thrive at Work Wellbeing Programme Collaboration

Abstract

Background: Good employee health and wellbeing is of key importance to employers and the economy. The workplace can serve as a setting for health and wellbeing promotion. Financial incentives may encourage employers to invest in employee health and wellbeing. The aim is to evaluate the effectiveness of health and wellbeing financial incentives offered to small medium enterprises in the West Midlands, UK.

Methods: A cluster randomised controlled trial was designed to evaluate the effectiveness of a workplace health and wellbeing initiative with or without monetary incentives. We will evaluate the effectiveness of the financial incentive using a mixed methods evaluation approach.

Discussion: The trial will help establish whether small-medium enterprises will improve their health and wellbeing offer and achieve higher employee awareness and participation in the offer in response to a monetary wellbeing incentive.

Trial registration: AEARCTR-0003420, registration date: 17.10.2018, retrospectively registered.

Keywords: Health and wellbeing, Workplace, Employment

Background

Good employee health and wellbeing is of key importance to employers and the economy. For example in the workplace, 6.5 m employees reported musculoskeletal problems in 2008 and this is predicted to rise to 7 m employees by 2030 [1]. This equates to around 1 in 8 employees in England reporting having a musculoskeletal problem. In the West Midlands, the average number of sick days per person per year is 6.9. This indicates that up to 1.5 days per person per year could be lost due to MSK in the West Midlands. Similarly the impact of mental ill health in the West Midlands is significant; overall mental ill health costs the West Midlands an estimated £12.6 bn annually, equivalent to £3100 per population head, per year. Employment costs as a result of mental ill health represent 31.5% of this total at £3.9bn. These costs include absence and presenteeism, as well as the cost of worklessness [2].

As a setting for health promotion, workplaces enable access to large groups of people to promote health and wellbeing. The European Agency for Safety and Health at Work provides evidence that workplace health promotion can lead to cost savings for organisations by reducing staff turnover and sickness absence, and is a valuable complement to health and safety programmes [3]. The European Agency for Safety and Health at Work recommend that a range of options are provided to businesses to implement workplace health promotion. Despite this evidence, many businesses find it hard to invest in the health and wellbeing of their employees, or do not invest at all; problems that are especially true for small and medium size enterprises (SMEs). There has

* Correspondence: Lena.al-khudairy@warwick.ac.uk
Division of Health Science, Warwick Medical School, Warwick University, Gibbet Hill Road, Coventry CV4 7AL, UK

© The Author(s). 2019 **Open Access** This article is distributed under the terms of the Creative Commons Attribution 4.0 International License (http://creativecommons.org/licenses/by/4.0/), which permits unrestricted use, distribution, and reproduction in any medium, provided you give appropriate credit to the original author(s) and the source, provide a link to the Creative Commons license, and indicate if changes were made. The Creative Commons Public Domain Dedication waiver (http://creativecommons.org/publicdomain/zero/1.0/) applies to the data made available in this article, unless otherwise stated.

been considerable interest in the effect of financial incentives to stimulate change in the workplace environment, which have shown promising results in health care settings [4].

The increase in the last decade of schemes aimed at changing the health related behaviour of the public has been accompanied by evidence that even small incentives can positively influence choices [5–8]. Nationally there are two examples of using financial incentives to encourage employers to invest in employee wellbeing:

1. Commissioning Quality and Innovation (CQUIN) [9] - initiative to improve health and wellbeing of NHS staff where employers are financially penalised if they do not achieve wellbeing targets. However, the fiscal incentive programme is designed to be a reward model rather than a penalisation model and reach a more diverse (and hard to reach) audience with significant barriers to uptake.
2. Health, Work and Wellbeing Challenge Fund [10] - This was a Competitive grant scheme in 2010 for SMEs, coordinated by Department for Work and Pensions and informed by "Working for a healthier tomorrow" report [11], however given it was a Competitive grant process it appealed to SMEs already engaged in employee wellbeing with an understanding of its importance. The fiscal incentive programme was designed to reach the 'unconverted' SMEs. Furthermore outcomes for the funding were self-reported and no baseline measures were taken to compare results to.

Neither of the above examples has systematically measured the change in the employers' perceptions at the organisation behaviour, health beliefs or health behavioural change.

This will be the first cluster randomised controlled trial (cRCT) of its kind to test a financial incentive in such a diverse range of organisational sizes and sectors. We intend to engage a mix of industries across the West Midlands economy currently giving the best chance of a nationally replicable programme. Furthermore a financial incentive has not been part of previous wellbeing commitments for employers so there is little to no comparable evidence about what changes an organisation has made and the effect on employees. Varying degrees of incentive has also not been previously been explored.

This study provides a unique opportunity to build the evidence for incentives to improve employee health and might help in identifying the financial 'tipping point' for employers to engage in improving the health and wellbeing of their employees. It will also build further understanding of the barriers and enablers for effective interventions aimed at employees. The intervention was designed and the research team was commissioned to carry out an independent evaluation, combining three interrelated aspects:

- Recruitment evaluation (to explore why some SMEs remained 'unconverted' and did not signed up to the trial)
- Impact evaluation (to systematically measure the change in the organisation behaviour) and
- Process evaluation (to understand how changes take place or why they do not occur).

This work can inform national policy regarding the effectiveness (or otherwise) of financial incentives and discussions about wider roll-out.

The aim is to evaluate the effectiveness of wellbeing financial incentives to SMEs in the West Midlands, UK. This primarily is to establish whether SMEs will improve their health and wellbeing offer and achieve higher employee awareness and participation in response to a financial wellbeing incentive. The evaluation also aims to assess whether financial incentives help and motivate SMEs overcome barriers when aiming to improve their wellbeing of their employees.

Methods

Trial design

The evaluation will employ a mixed methods design of a four-arm cross-sectionally cRCT with baseline measurements. The trial will encompass two levels of the intervention and a modified Solomon method whereby two control groups are formed only one of which contributes base line measurements. The evaluation will comprise three elements: recruitment evaluation, impact evaluation and process evaluation.

Study setting

The study will be carried out with SME's located in the West Midlands region, UK.

Eligibility criteria

SMEs (10–250 employees) that are located in the West Midlands Combined Authority footprint; receptive to implementing health and wellbeing behavior changes within workplaces (with or without monetary incentives); willing and able to provide organizational level data; willing and able to allow employees time to complete questionnaires and to allow senior executives to be interviewed. Employee inclusion: employees who are 16 years of age or older; who hold an employment contract with the employer; and who are willing to provide written consent.

Any issues around right of withdrawal of employees will be addressed by implementing the following:

- Participants will be informed about their right to withdraw from the study at any time before the interviews commence.
- Participants will be made aware that they can stop the interview if they wish to withdraw from the study at any time.
- If participants decide to withdraw their data post interview, every effort will be made to remove their data, however this may not always be possible if analysis and publication has already taken place. They will be informed that they will not have to give a reason for withdrawing and this will not affect their circumstances in any way.
- If choosing to withdraw from the study, any data collected before withdrawal will be deleted and quotes will be removed from any written reports if it is possible to do so – as above, if publication has already taken place, this will not be possible (but this will be made clear to participants as part of the consent process).

Interventions

The intervention is a monetary incentive that is designed to stimulate SMEs to improve the wellbeing offer to employees and promote the health of their workforce with respect to healthy behaviours such as mental health, healthy lifestyle and musculoskeletal health. The incentive will:

a) Vary in size depending on the number of employees and will be given at two levels (i.e. a smaller (50%) and larger (100%) financial allocation) and
b) Irrespective of the level, it would be paid in two instalments. The first instalment of 30% will be paid immediately following baseline assessments. The second instalment is based on how much of the Thrive at Work Bronze level Commitment has been implemented.

All SMEs (intervention and control) will receive a Thrive at Work Wellbeing Commitment Programme and Toolkit. This is a guideline that provides organisations with a structured method they can follow in order to improve the health and wellbeing of employees. Topics covered include mental health, musculoskeletal health, and healthy lifestyle. The Thrive at Work programme includes a supporting toolkit of available local and national resources that the company can draw on in their efforts to raise awareness of health issues among employees and to prompt employees to take action to improve their health and wellbeing. The Thrive at Work pack provides an opportunity for SMEs to attend quarterly network meetings that are designed to spread the best practice across SMEs in relation to the Thrive at Work Wellbeing Commitment Programme and Toolkit.

The toolkit in relation to the incentive

The toolkit is an inexpensive method than can be implemented at scale. The hypothesis that this study aims to test is that the uptake of toolkit can be increased by a monetary incentive.

Outcome measures

The primary aim of the study is to establish whether SMEs will improve their health and wellbeing offer in response to a monetary wellbeing incentive targeted to the organisation not the individual. However, asking employers to assess themselves against this outcome is open to inaccurate self-assessment. Hence the need for an external assessment in which individual employees will be interviewed. The NHS Commissioning for Quality and Innovation (CQUIN) indicator specification [12] indicates the following question in the annual staff survey:

'Does your organisation take positive action on health and wellbeing?'

The sample size was calculated on the basis of this question.

Additional outcomes

Additional outcomes will be collected from both employers and employees. Therefore, we will have two sets of results presented by status (employer vs employee). Questions will cover each of the key theme areas (i.e. lifestyle, musculoskeletal, mental health). Employee wellbeing will also be measured using the four main questions used by the Office for National Statistics to monitor national wellbeing [10]. See Additional file 1 for full questionnaire.

A number of the employee level questions will align with those asked to the employer in order to assess consistency between the two.

These questions cover the following:

- Awareness of information provided by the employer (directed to employer and employee)
- Likelihood of taking part in the health and wellbeing offer provided by the employer (directed to employer and employee)
- Improvement efforts carried by the employee (directed to the employee)
- Provision of activity / services provided by the employer (directed to employer and employee)
- Utilisation of activity/services by the employee (directed to employer and employee)

Additional measures for the employer will include:

- Policies
- Regulations

Recruitment evaluation

The aim of the recruitment evaluation is to understand the reasons for non-participation in the trial thereby providing insight into the barriers, motivators and enablers that influence the success or otherwise of recruitment to the programme. SMEs in the West Midlands who had the opportunity to participate but did not do so will be approached to provide information on their reason for non-participation in order to inform future studies and policies, and so that any factors that may potentially affect drop out are considered in relation to the main study.

The recruitment evaluation questions are as follows:

1. What (if any) aspects of the programme influenced organisations' decision to participate or not participate?
2. What (if any) aspects of the trial design influenced organisations' decision to participate or not participate?
3. What (if any) aspects of the recruitment process influenced organisations' decision to participate or not participate?
4. What other reasons (if any) influenced organisations' decision to participate or not participate?

Process evaluation

Intervention logic

A logic model (see Additional file 2) was developed in an internal workshop to capture the essential elements of the intervention and mechanisms through which the intervention is meant to work. The top level (in green) relates to the inputs to the trial provided by the intervention provider based on the Thrive at Work guidelines (website and toolkit) and provision of the financial incentive in SMEs in the intervention groups. The next level (in orange) relates to the activities that might be undertaken as a result of these inputs, for instance a health needs assessment being undertaken in response to the Thrive at Work toolkit. The next level (in red) relates to the outputs produced by these activities, such as employers' increased knowledge and awareness as a result of additional support being put in place. The following layer (in blue) relates to outcomes i.e. more long-term changes taking place as a result of these outputs, for instance employees making healthier lifestyle choices. The final two layers relate to the goals (in turquoise) and long-term goals (in purple) of these changes. To give an example, one of the goals of the trial is to encourage a fitter, more resilient workforce, but this also feeds into a longer-term goal of reducing public spending on health and welfare.

The second model (see Additional file 3) focuses on the intervention in the form of the financial incentives and describes the theory behind the expected effects (outputs) of the intervention.

These models provide the basis for development of the main process evaluation questions briefly outlined below.

Process evaluation questions

We anticipate that the process evaluation, will cover eight high level topic areas. Within each area, we will design questions to identify the main mechanisms which drive behaviour change by employers and employees and the factors which act as motivators, barriers and enablers. As such, the process evaluation will complement the impact (quantitative) evaluation: it will examine how the incentive may interact with the Thrive at Work Commitment and toolkit and identify the mechanisms by which the incentive does or does not bring about change. The eight topic areas covered are shown below, with indicative examples of issues we propose to explore within each:

1. Were the fiscal incentive, Thrive at Work Commitment and network meetings implemented with a high level of fidelity?
2. How effective have communications about the intervention been (from West Midlands Combined Authority to employer and from employer to employee)?
3. How have employers and employees identified what needs to change in terms of health and wellbeing activities and outcomes?
4. What health and wellbeing offer was made by the employer before and since their participation in the trial?
5. Have employees' intentions or behaviour changed towards the health and wellbeing offer?
6. To what extent (if at all) and how have the initial grant (and prospect of further payment) triggered commitment and activity on the part of the employer?
7. To what extent and how have the Thrive at Work Commitment and toolkit been effective in facilitating behaviour change among employers and employees?
8. How, if at all, have the network meetings helped employers enhance their health and wellbeing offer?

Participant timeline

Baseline assessments will be collected from three arms (I^1, I^2, C^1) but not one of the control arms (C^2). Post-intervention quantitative assessment will be collected at 12 months from the four trial arms (I^1, I^2, C^1, C^2). The recruitment evaluation from clusters that decline will take place as soon as possible after recruitment closes and while memories of reasons for non-

participation are still fresh. Process evaluation will collected throughout the trial period, in particular at post-randomisation (I^1, I^2, C^1), midline (I^1, I^2) and post-intervention (I^1, I^2, C^1, C^2). Figure 1 illustrates trial and participant timelines.

Sample size

Power calculations are conducted around the principle of observing "statistically significant" differences between study arms. "Statistical significance", however, is not a concept typically used in Bayesian analyses, nor is it the appropriate consideration here for determining the effectiveness of our intervention. We conduct instead an "assurance analysis" to determine the probability of estimating an effect size to within a given degree of certainty with the data generated by the trial [13]. This has a more natural interpretation and allows us to take into account our prior uncertainty over effect size. In this case, the appropriate level of certainty is that which ensures a negligible probability of the sign of the effect (i.e. positive or negative) being incorrectly inferred. Given that the assurance calculations are intractable mathematically, we simulate a large number of trials and analyses.

Overall, 132 SMEs are planned to participate in the trial, and they will be allocated equally between arms, i.e. 33 per trial arm. We aim to interview a random sample of ten employees per SME. For the purposes of the design evaluation and generation of simulation data we consider informative priors (similar to prior beliefs about effect size in frequentist power calculations albeit with uncertainty), as distinct from the analysis priors to be used in the analysis stage and model estimation. The treatment effects for the 50 and 100% incentive arms were modelled as 1.3 ± 0.1 and 1.5 ± 0.1, respectively. The baseline was assumed to be between 10 and 50%. For cRCTs such as this, the variance can be partitioned into three components: between cluster, within cluster-between period, and within-individual. The 'power' of the trial design depends on the intra-cluster correlation coefficients (ICC) (both between and within periods) and the cluster autocorrelation (CAC), which captures how similar cluster outcomes are over time. We also assumed the within-period ICC (the proportion of variance attributable to the cluster level) would lie between 0.02 and 0.1 uniformly and the CAC would be uniformly distributed between 0.75 and 1. Finally, we simulated the measurement effect to be, as a relative risk ratio, between 0.9 and 1.1.

Based on the design of the trial, we determined the probability that there would be at least a 95% posterior probability that each treatment effect (as an odds ratio) would be > 1 and hence that the absolute difference between the treatment effects would be > 0. The respective probabilities for the 50 and 100% treatment conditions were 70 and 79%, and the probability for the difference was $> 99\%$. Therefore, we consider the sample size to be sufficient. For comparison, a "classical" power calculation with 5% type I error probability and values at the mean values of the parameters stated above for a two-arm trial gives a power of 68 and 92% for the two treatment conditions respectively [14].

Recruitment

The West Midlands Combined Authority has established a pivotal role in the West Midlands. "Market warming" prior to the trial, essentially early notification and discussion about the intended intervention, found and generated high interest in the programme amongst SMEs.

SMEs were recruited through a planned and targeted emailing campaign via key public and private sector leaders and colleagues and 'cascaders' known to hold lists of stakeholders and businesses in the midlands region.

Cascaders are recognised as being able to provide access to SMEs that are affiliated or associated with them and more likely to sign up through this relationship/introduction. (For example, Birmingham Chamber of Commerce can identify 16,000 SME recipients for information they already cascade).

Cascaders were prepared for their role through a series of face to face meetings and conference calls. This activity allowed the project to understand the issues faced by cascaders in emailing information out, for example availability of information technology, size of documents, format preferences, timing etc. Campaign Monitor (an online marketing tool) was used to push emails to cascaders to allow them to then forward information and a web link to the Thrive at Work Wellbeing Trial and application form.

The cascade campaigns were supplemented by:

- A major Twitter, (Mayor, Sean Russell, West Midlands Combined Authority), LinkedIn and Facebook campaign with updates coinciding with cascade campaign dates.
- West Midlands Combined Authority website updates and showing 'news' on Thrive at Work Wellbeing Trial. This was updated regularly.
- Personally calling businesses selected to cover off geographical and industry type spread requirements.
- Asking all West Midlands Combined Authority staff to support recruit in through their networks.
- Using expertise from West Midlands Combined Authority Marketing and Communications Teams and using their networks and links to access applicants or more 'cascaders'.
- Creating videos to support website content and Twitter, Facebook and LinkedIn campaigns.

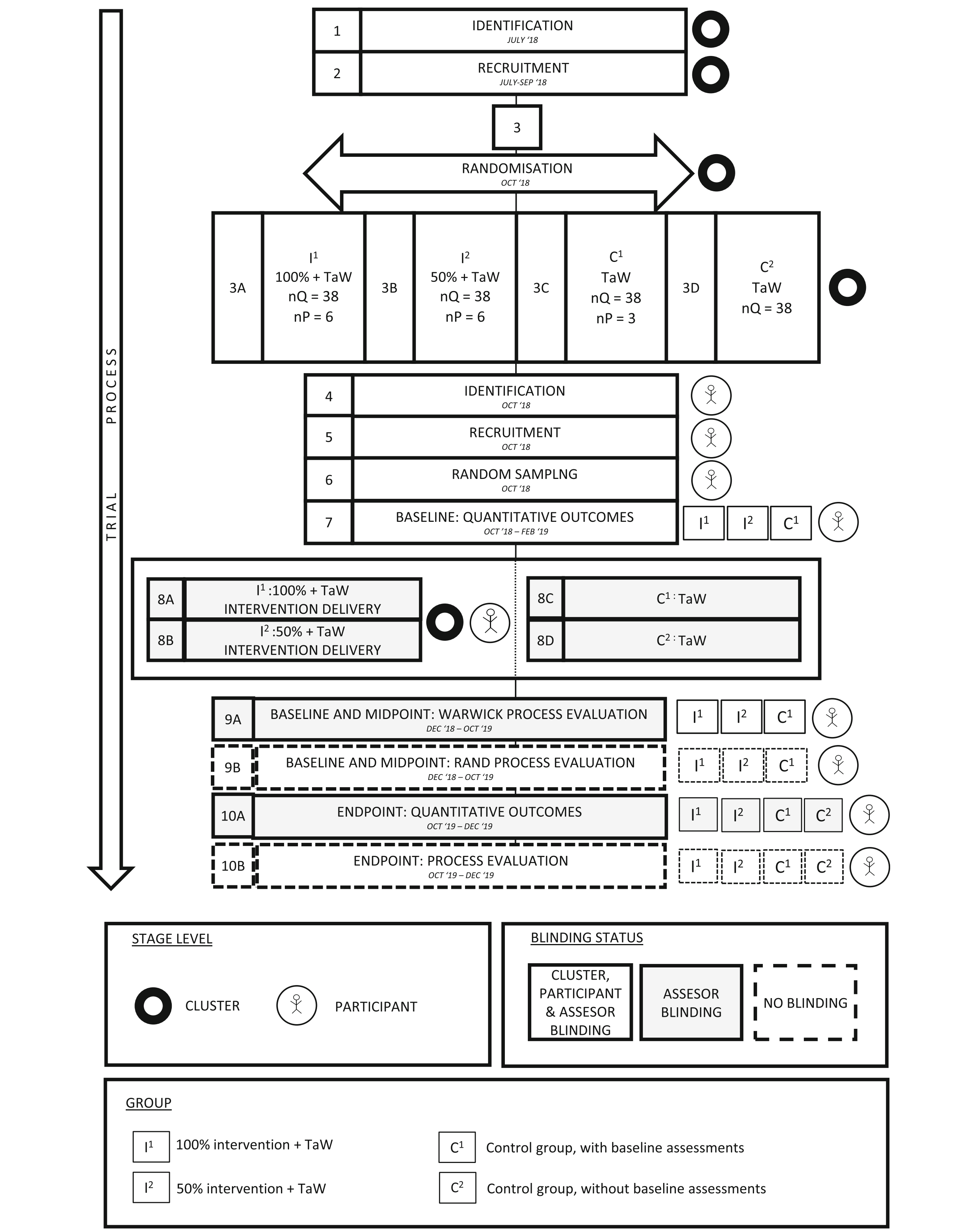

Fig. 1 Timeline cluster diagram of the study

Progress was assessed weekly, and recruitment activity was adjusted accordingly. Enrolment was carried out over a two and a half month period (July 2018–September 2018).

Randomisation

Covariate-constrained randomisation procedure

Cluster randomised controlled trials (cRCTs) often face the issue of only having a relatively small number of participating clusters, which can lead to large imbalances in covariates across trial arms with straightforward randomisation techniques. Covariate-constrained randomisation is an allocation technique that ensures balance across trial arms in confounders that are specified a priori. The procedure for conducting a constrained randomisation is: (i) specify the relevant covariates and collect information on these for each cluster; (ii) enumerate all or a large number of possible randomisation schemes; (iii) remove duplicate randomisation schemes; (iv) select a candidate subset of schemes using an appropriate balance metric; (v) randomly select one of the candidate schemes [15]. A number of articles have explored the performance of constrained randomisation procedures, and a growing number of cRCTs have used it in practice [16]. However, almost exclusively these have focussed on two-arm trials. Below we provide a procedure for extending covariate-constrained randomisation to multiple arms on the basis of work in similar techniques such as re-randomisation [17].

The two key confounding covariates we identified that will form the basis of the balancing are SME size and SME industry type. SME size is the number of employees the SME has, which likely impacts upon how or if workplace health and well-being initiatives are implemented. The type of industry the SME is involved with is known to be related to the socio-economic status and social class of the employees, which may have an impact on attitudes to health and well-being. We classified the SMEs into three broad categories on the basis of the UK Standard Industrial Classification of Economic Activities 2007 (SIC): [18] manual and secondary sector (including manufacturing, construction, and energy supply; SIC Sections C-F); service and tertiary sector (including professional services, finance, and information and communication; SIC Sections G-N, R, and S), and social and public sector (including education, health, and public administration; SIC Sections O-Q). We generated 100,000 randomisation schemes and removed any duplicates. We randomly selected among the top 1% of allocation schemes according to the balance metric.

Balance metrics for constrained randomisation, such as the imbalance score [19], compare two arms. For multi-arm cRCTs, test statistics used in multivariate analysis of variance (MANOVA) provide a good univariate measure of covariate balance [17]. The balance metric we use is Wilks' Λ, which is described in the Appendix. Larger values of Wilks' Λ correspond to better covariate balance. We select the scheme with the highest value. Within each SME we randomly select ten employees. This selection is completely random with no constraint. Code for conducting the procedure is provided in the Additional file 4.

Implementation

Enrolment was carried out by the West Midlands Combined Authority team. Allocation was carried out by a statistician (SW) at Warwick Medical School to randomly allocate clusters to one of the four arms:

1. Intervention (1) baseline and post intervention assessment: 100% of the monetary incentive + Thrive at Work Commitment and Toolkit
2. Intervention (2) baseline and post intervention assessment: 50% of the monetary incentive + Thrive at Work Commitment and Toolkit
3. Control (1) baseline and post intervention assessment: Thrive at Work Commitment and Toolkit
4. Control (2) post intervention assessment only: Thrive at Work Commitment and Toolkit

Blinding (masking)

To avoid ascertainment bias, baseline bias assessments will be carried out prior to the start of the intervention so participants will not know which groups they have been randomised to when baseline assessment is carried out. SMEs will be informed of their random allocation post baseline assessments. This information will come from the West Midlands Combined Authority in a letter sent out via email after baseline assessment.

While randomisation provides strong protection against selection bias, we were concerned to avoid observer bias that could result if observers were aware of group allocations. To minimise this risk we decided to allocate separate observers, deployed from Warwick University, to conduct baseline vs. follow-up observations. Quantitative and qualitative data collection will be undertaken by Warwick University researchers (4 researchers) visiting the workplaces and interviewing employers and employees. Quantitative outcomes will be face-to-face with iPad tablets, whereas process evaluation will take place over the phone and/or face-to-face using a topic guide. Process evaluations follow quantitative evaluations so that the latter cannot contaminate the former. To minimise observer bias there are three methods proposed:

1. Those who collect process data will not collect impact data from the same institution.

2. Data collectors undertaking interviews with employees will not be informed of a companies' allocated group.
3. Outcomes data collectors will be asked of their suspicions about which group the SME they are collecting data from is in. This can then be compared to the actual groups the SMEs are in, to see how good the observer blinding has been. We will use a separate team of telephone interviewers in Cambridge (RAND) to conduct interviews with employers as part of the process evaluation (see below). These interviewers will not be blind to the allocation as they will ask questions about how the employers in the Intervention groups have used the money, and the value and impact of the network meetings. We will brief these interviewers not to share their knowledge with the team conducting interviews with employees for the process evaluation (who will thus remain blind to the allocation).

Data collection

Quantitative outcomes (random sample selection of employees for impact evaluation)

Warwick Medical School will receive an anonymised coded list of employees from the West Midlands Combined Authority. Warwick Medical School will select a random sample of employees for baseline assessment (in ¾ of the groups) and outcomes assessment, a minimum of 10 employees per SME. The random sample will be sent back to the West Midlands Combined Authority for decoding. West Midlands Combined Authority will send a list of employees (provided consent to West Midlands Combined Authority) to outcome assessors (Warwick University) to arrange for data collection. Having the West Midlands Combined Authority (an independent institute) code the SMEs will secure blinding of evaluators (outcome assessment, analyses and findings).

Quantitative outcomes will be collected by qualified researchers (all hold a postgraduate degree). After arranging a suitable time for data collection, researchers will visit workplaces and interview employers and employees to complete an electronic questionnaire face to face. All the researchers in this project will use standardised methods of data collection in order to reduce potential errors and ensure comparability. Researchers were trained following a data collection protocol developed for this evaluation to ensure that the data are collected systematically.

Researchers will be equipped with tablet devices (Apple iPads), which run the Mac OS system. Devices are password protected and marked with a unique identification number. Qualtrics software was used to produce an electronic questionnaire. For each device Qulatrics was set-up with the support of the information technology department at Warwick University.

The questionnaire was tested for length and content in three SMEs that did not meet the inclusion criteria of this trial. The questionnaire was then revised prior to being used in the field.

Primary and secondary quantitative outcomes will be collected at two time points over a 3 months period. Post-randomisation pre-intervention assessments: SMEs will be randomised to one of the four arms but will not be informed of allocation until post-baseline assessments.

Recruitment evaluation

The recruitment evaluation will use qualitative methods to understand the reasons for non-participation. There are two main strands to the recruitment evaluation, which will operate consecutively:

1. A review of the recruitment strategy and materials produced by the West Midlands Combined Authority and interviews with 3 to 5 key personnel, possibly including cascaders (i.e. representatives from organisations who disseminated information about the trial to SMEs in the region).
2. Semi-structured, telephone interviews with 10–20 non-participating SMEs. Due to the nature of the sample (i.e. employers who are relatively disengaged and/ or time poor), it is unlikely that more than 20 will be willing to take part in an interview, but this would be the maximum number of interviews if this were to occur. The recruitment to the evaluation will be sensitive to the fact that this group of SMEs are likely to be relatively disengaged. The interviews will last between 5 and 30 min and will be arranged at a time to suit respondents. To maximise response rate, flexibility will be built into the interview guide, allowing employers to participate in a relatively short discussion if they are unwilling or unable to speak for longer.

The sample will include SMEs who received information regarding the trial but chose not to participate, and those who agreed to take part but pulled out before randomisation (if any). These SMEs will be approached and asked to provide information on their reasons for non-participation, and any factors that might have changed their decision, in order to inform future studies and policies. This will also allow us to identify any factors that may potentially have affected drop out so that their impact can be taken into account in relation to interpreting the results of the main study.

Process evaluation

Process evaluation data will be collected using a combination of semi-structured interviews with employers and employees; document review (particularly of the Thrive at Work commitment and toolkit and other

communication tools produced by West Midlands Combined Authority); direct observation and review of attendance records at the quarterly network meetings; and review of supporting evidence, such as the statements of spend produced by participating SMEs.

Detailed qualitative interviews will be undertaken in a sample of SMEs per trial arm (see Additional file 5). These will be selected to cover a spectrum in terms of size (with matched groups from each trial arm) and industry sector.

We will aim to interview - a minimum of 3 employees in the smallest SMEs, and a maximum of 6 in the largest (see Additional file 5), ideally with a different mix of individuals at each stage of the process evaluation. However, we will also be pragmatic about which employees we interview depending on availability on the day of our visit and in order to minimise disruption to the business activities of SMEs. The interviews will build on the logic model (see above) and focus on the barriers and facilitators of change at the organisational and individual level.

We want to interview SMEs from the control to understand what influences behaviour change among those employers receiving only the Thrive at Work commitment and toolkit and having the opportunity to attend network meetings. It will be instructive to see how the mechanisms of change and barriers for the control groups differ (if at all) from those observed in SMEs who do receive a financial incentive.

Separately, we will conduct telephone interviews with the Thrive at Work lead (i.e. official(s) sponsoring or overseeing participation in the trial) at each of these SMEs. These interviews will provide data on how the initial monetary incentive payment has been used by employers and what activities it has enabled, and on why employers chose to use it as they did. They will also enable us to understand: employers' perceptions of the value and impact of the Thrive at Work commitment and toolkit; to what extent the final monetary incentive payment is motivating activity to achieve Thrive at Work accreditation; whether or not the employer has attended quarterly network meetings; and if so, what value these meetings had for them.

Qualitative data will be collected through a combination of face to face and telephone interviews carried out by qualified researchers (all hold a postgraduate degree) following an interview guide.

Data management and monitoring

Impact evaluation: data will be exported from password protected University managed data collection tools (tablets) and stored electronically on University managed password protected computers at the University of Warwick. Once data are exported from tablets they will be deleted from this device. Passwords will be longer than 8 characters and will include an uppercase letter, lower case letter and a symbol. These will only be accessible by researchers of the study. Any paper based reports or data related to this project will be stored in locked filing cabinets. Participant data will be kept for 10 years once created according to the University of Warwick's guidelines.

We will classify data according to the standards used by the University of Warwick as follows:

- Public: No risk - confidentiality is of no particular significance to this information.
- Protected: Low risk - inappropriate disclosure would have minimum significance.

**Classification**	**Public**	**Protected**	**Restricted**	**Reserved**
**Risk**	**None**	**Low**	**Medium**	**High**
Access	May be viewed by anyone, anywhere in the world	Available only to specified authorised University of Warwick members (Workplace evaluation project)	Available only to specified authorised University of Warwick members (Workplace evaluation project)	–
Personal information	Anonymised information (results of the project that contain no identifiable information, findings of the project such as % of employees answering yes to *Does your organisation take positive action on health and wellbeing*)	Academic qualification of employee, working pattern (full-time vs part-time)	Employee name, age, sex, ethnicity	–
Non-personal information	Nature of business (public, private, voluntary), number of employees in the business, % of males in the business, % females in the business	Organization address	–	–

Identifiable data must only be shared via a shared drive and must be encrypted when not in use. The anonymised data can be stored unencrypted but must not be shared with anyone outside of the project without the permission of the project management members. Data may also not

be shared using USB stick, or other portable media, unless the media device is password protected.

The approach to data management aims to be GDPR-compliant. For example, all participants are informed about what will happen to their data in a Participant Information Sheet. The Qualtrics software used to conduct the impact evaluation allows for individuals' responses to be modified and deleted upon request. Personal data will not be shared in an identifiable format. The devices are encrypted, and we have secure servers for storing the data. Quality assurance checks will be done of the data to ensure completeness and accuracy. The approach to data management has been discussed with and approved by Warwick University data management teams.

Recruitment evaluation and process evaluation: we will not need to store any personally identifiable data. We will train the research team about our requirements and how to treat any sensitive data that might be collected in the process evaluation interviews with employers and employees. Transcripts and audio files from the interviews are confidential and these data will all be classified as *Reserved*. Any audio recordings will be used by interviewers only for the purposes of assisting with the completion of interview notes and will then be deleted.

The final cleaned anonymized data set and anonymized findings will be made available to collaborators. Project members are expressly forbidden from sharing data collected in this project via email. Similar to the impact evaluation, the approach to data management in the recruitment and process evaluation aims to be GDPR-compliant. It has been discussed with and approved by the Data Protection Officer at RAND Europe.

We will use RAND's process to quality assure the final evaluation report, covering all three strands of the evaluation. Quality assurance of all outputs of the project will be undertaken jointly by Warwick University and RAND. RAND will lend its quality assurance procedures and standards for the purpose of the two elements of this evaluation, namely:

– Recruitment evaluation
– Process evaluation

Warwick University will follow standard procedures for checking the quality of the impact evaluation data such as reviewing each questionnaire post completion following a standardised protocol and random spot checking of the dataset.

Analysis plan

Quantitative outcomes

Descriptive data for continuous measures will be presented as means±SD. Descriptive data for categorical measures will be presented as numbers and percentages.

We will conduct a Bayesian constrained regression-based analysis of the trial data that incorporates the possibility of an additive measurement effect and time effects. The constrained regression analysis assumes that there are no systematic differences between clusters at baseline and hence no covariates are included in the model, which reflects the constrained randomisation procedure described above. The model includes treatment effects for each treatment condition, an effect for if the cluster has been measured prior to the time of observation, a cluster random effect, fixed effects for each time point, and a random cluster by time point interaction. We will use a log-binomial model as the interest lies in relative risk effects, which this model's parameters provide. The full specification is provided in the Additional file 6.

We opt for a Bayesian approach to allow for the incorporation of prior uncertainty, facilitate computation of a complex hierarchical model, and provide a more intuitive interpretation of parameter estimates, which naturally incorporate into decision analyses such as economic evaluations. We will assign "weakly informative" priors to the model parameters, which provide little information on the parameter value but ensure regularisation of the model and facilitate computation. In particular, we will use normal distributions with a standard deviation of ten for parameters in the linear predictor and half-standard Cauchy priors for the hyper parameter variance terms [13, 14].

Recruitment evaluation

Following the completion of the interviews, data will be synthesised and consolidated into a final report. Qualitative data will be analysed thematically, drawing out key themes and findings. Feedback from employers will be fully anonymised and presented at an aggregate level. Where appropriate (and non-identifying), the analysis will be supported by verbatim quotes from respondents.

Process evaluation

The process evaluation will generate large amounts of interview data. Qualitative data analysis - and within this - thematic analysis will be used to sort and examine the data to gain a good understanding what they contain. It will be used to identify recurring themes (that reflect specific patterns or meaning found in the data), to categorise these by applying codes to a portion of data, to describe the range of factors, attitudes, behaviours, etc., and to explore and explain in more detail how the intervention worked in practice. The interview transcripts and notes will be analysed using specialised software for qualitative data analysis (NVivo). A coding framework will be developed based on the interview guides, logic model and the Theoretical Domains

Framework in order to systematically code and analyse portions of data. At the same time we envisage that the framework will allow some flexibility to capture issues emerging directly from the data and that it will evolve over time to address issues coming into light in the mid- and end-point of the trial implementation.

Data monitoring

Described previously under data management and monitoring.

Harms

Researchers will complete questionnaires and conduct qualitative interviews in a sensitive, non-judgmental manner. Research assistants are trained researchers and will adhere to research ethics and standards. Quantitate evaluation will not be collecting any sensitive information. However, researchers may need to communicate disclosures outside the research team if any participant states anything that may place themselves or anyone else at harm. In addition, researchers can provide participants contact details of local and national services if something sensitive was disclosed. RAND will brief the research team appointed by Warwick about their requirements and how to treat any sensitive data that might be collected in the process evaluation interviews with employers and employees.

Protocol amendments

Protocol modifications will be immediately reflected on the trial registry (AEARCTR-0003420) and will be communicated to relevant parties such as The Work and Health Unit, research team, recruited businesses and journals as applicable.

Confidentiality

- Participants will be assigned ID numbers so no identifiable information will be published.
- Names or identifiable characteristics will not be used in interim reports or publicly available reports or publications.
- If names or identifiable characteristics are used by participants in qualitative interviews, these will be changed within the transcription to avoid identifying individuals.
- Telephone interviewers will conduct calls in private rooms to ensure confidentiality, interviewees will be encouraged to participate in private.

Storage of personal information is described above under data management and monitoring.

Dissemination policy

SMEs will be offered a copy of the report. This project will be published as interim and final reports to the funder (The Work and Health Unit). It will also be written up as manuscripts for publication in a peer-reviewed academic journal. The findings will be disseminated to Warwick University, RAND and West Midlands Combined Authority through written and oral communications.

Discussion

The trial is designed to evaluate the effectiveness of a fiscal incentive in improving the awareness and uptake of health and wellbeing offer at SMEs across the West Midlands. However, there are challenges in the design and approach considered.

The limited funding horizon dictates the type of endpoint that could be measured or might reasonably be expected to change in response to the intervention. Thus we did not plan to measure the actual health of employees, say in terms of disease rates. We think that expecting an intervention of this scope to boost productivity is rather hopeful. We also note that the main endpoints are necessary, but not sufficient, markers for improvements in health and productivity. Thus, we proceeded on the basis that if the short-term endpoints captured in this study changed in a positive direction, then it would be possible, given resources, to model potential effects on health.

Any results from the intervention will only generalise to those SMEs and employees eligible to take part, although broader lessons may be learned that can generalise to other similar SMEs.

Researchers are increasingly expected to collaborate with policy-makers. This is for the very good reason that robust, prospective designs usually require such collaboration. However, policy-makers often have an imperative to expedite the promulgation of interventions. In such circumstances the researcher has to meet the policy-maker in the middle. Such was the case here, and we have tried to adapt to this requirement with a design that maintains rigour, but which is achievable in the demanding timescale. Nevertheless, this trial will provide robust evidence on the effect of monetary incentives on health and wellbeing in the workplace.

Additional files

Additional file 1: Questionnaire for quantitative outcomes (employer and employee each administered separately). (DOCX 16 kb)

Additional file 2: Logic model of the Wellbeing Premium Programme or Thrive at Work used for process evaluation to capture the essential elements of the intervention and mechanisms through which the intervention is meant to work. (DOCX 606 kb)

Additional file 3: Logic model of the Wellbeing Premium Programme or Thrive at Work used for process evaluation that focuses on the intervention in the form of the financial incentives. (DOCX 84 kb)

Additional file 4: Description of the constrained randomisation balance metric. (DOCX 12 kb)

Additional file 5: Estimated number of interviews per arm for process evaluation. (DOCX 12 kb)

Additional file 6: Specifications of statistical analysis planned for quantitative outcomes. (DOCX 13 kb)

Abbreviations

CAC: Cluster autocorrelation; CQUIN: Commissioning for Quality and Innovation; cRCT: Cluster randmonised controlled trial; MSK: Musculoskeletal; NHS: National health services; nP: Number of organisations for process evaluation; nQ: Number or organisations for quantitative assessments; SMEs: Small medium enterprises; TaW: Thrive at Work

Acknowledgements

We would like to acknowledge Mr. Andrew Rudge from the West Midlands Combined Authority for supporting this project.
We would like to acknowledge the Collaboration for Leadership in Applied Health Research and Care West Midlands – NIHR.
Thrive at Work Wellbeing Programme Collaboration:
Yasmin Akram, West Midlands Combined Authority, 16 Summer Lane, Birmingham, B19 3SD, United Kingdom. Yasmin.Akram@wmca.org.uk
Lena Al-Khudairy1, Warwick Medical School, Warwick University, Gibbet Hill Road, Coventry, CV4 7AL, United Kingdom. Lena.al-khudairy@warwick.ac.uk
Karla Hemming, Birmingham University, Edgbaston, Birmingham, B15 2TT, United Kingdom. k.hemming@bham.ac.uk
Joanna Hofman, RAND Europe, Westbrook Centre, Milton Road, Cambridge, CB4 1YG, United Kingdom. jhofman@rand.org
Laura Kudrna, Warwick Medical School, Warwick University, Gibbet Hill Road, Coventry, CV4 7AL, United Kingdom. Laura.Kudrna@warwick.ac.uk
Richard Lilford, Warwick Medical School, Warwick University, Gibbet Hill Road, Coventry, CV4 7AL, United Kingdom. R.J.Lilford@warwick.ac.uk
Madeline Nightingale, RAND Europe, Westbrook Centre, Milton Road, Cambridge, CB4 1YG, United Kingdom. mnightin@rand.org
Rob Prideaux, Senior Research Leader, RAND Europe, Westbrook Centre, Milton Road, Cambridge, CB4 1YG, United Kingdom. prideaux@rand.org
Sean Russell, West Midlands Combined Authority, 16 Summer Lane, Birmingham, B19 3SD, United Kingdom. sean.russell@wmca.org.uk
Alex Sutherland, RAND Europe, Westbrook Centre, Milton Road, Cambridge, CB4 1YG, United Kingdom. asutherl@rand.org
Ivo Vlaev, Warwick Business School, University of Warwick, Coventry, CV4 7AL, United Kingdom. Ivo.Vlaev@wbs.ac.uk
Christian van Stolk, RAND Europe, Westbrook Centre, Milton Road, Cambridge, CB4 1YG, United Kingdom. stolk@rand.org
Samuel Watson, Warwick Medical School, Warwick University, Gibbet Hill Road, Coventry, CV4 7AL, United Kingdom. S.Watson.1@warwick.ac.uk

Funding

This trial was funded by The Work and Health Unit – United Kingdom (governmental funding). The study funder reviewed the study design but had no role in the writing of this manuscript or the decision to submit this manuscript for publication. The study funder will have no role in the collection, management, analysis and interpretation of data. Contact details workandhealth@dwp.gsi.gov.uk

Availability of data and materials

Not applicable.

Authors' contributions

YA: contributed to the conception of the evaluation, evaluation design, drafting the trial protocol and final amendments of the version to be published. Read and approved the final manuscript. LA-K: contributed to the conception of the evaluation, design of quantitative data collection tools and quantitative data management, ethics, registration of the trial, drafting the trial protocol and final amendments of the version to be published. Read and approved the final manuscript. KH: provided guidance on the trial protocol and critically evaluated the intellectual content of the protocol. Read and approved the final manuscript. JH: contributed to the conception of the evaluation, design of data collection tools, drafting the trial protocol and final amendments of the version to be published. Read and approved the final manuscript. LK: contributed to ethics, design of quantitative data collection tools and quantitative data management, and final amendments of the version to be published. Read and approved the final manuscript. RL: Primary investigator of Warwick University work package, contributed to the conception of the evaluation, designed the evaluation and critically evaluated the intellectual content of the protocol. Read and approved the final manuscript. MN: contributed to the conception of the evaluation, design of data collection tools and drafting the trial protocol. Read and approved the final manuscript. RP: contributed to the conception of the evaluation, design of data collection tools, drafting the trial protocol and final amendments of the version to be published. Read and approved the final manuscript. SR: Primary investigator of the West Midlands Combined Authority work package, contributed to the inception of the evaluation, lead the inception of the evaluation, contributed to the evaluation design, drafting the trial protocol and final amendments of the version to be published. Read and approved the final manuscript. AS: Primary investigator of the RAND Europe work package, contributed to the inception of the evaluation and the trial design. Read and approved the final manuscript. IV: contributed to the behavioural components of the study. Read and approved the final manuscript. CS: contributed to the inception of the intervention and evaluation. Read and approved the final manuscript. SW: contributed to the design of the evaluation, sample size calculation, randomisation, quantitative sample selection and critically evaluated the intellectual content of the protocol. Read and approved the final manuscript.

Ethics approval and consent to participate

An ethics submitted for the evaluation (quantitative and qualitative) was submitted to The Biomedical & Scientific Research Ethics Committee (BSREC) of Faculties of Medicine and Science. The application underwent peer approval and has been accepted (REGO-2018-2230).
In line with basic ethical principles, we will ensure that all SMEs and employees involved in the evaluation will participate voluntary with written informed consent and can withdraw from the study at any time. All participating SMEs within the West Midlands region should provide consent (collected by the West Midlands Combined Authority) for the study to be carried out within their organisation.

Consent for publication

Not applicable.

Competing interests

The authors declare that they have no competing interests.

Publisher's Note

Springer Nature remains neutral with regard to jurisdictional claims in published maps and institutional affiliations.

Received: 6 December 2018 Accepted: 22 February 2019
Published online: 02 May 2019

References

1. Vaughan-Jones H, Barham L. Healthy work: challenges and Apportunities to 2030: British United Provident Association; 2009.
2. Newbigging K. Mental Health in the West Midlands combined authority. Birmingham: University of Birmingham; 2017.
3. Hassard J, Wang D, Cox T, Muylaert K, Namysł A, Kazenas A, et al. Motivation for employers to carry out workplace health promotion: literature review. 2012.
4. Flodgren G, Eccles MP, Shepperd S, Scott A, Parmelli E, Beyer FR. An overview of reviews evaluating the effectiveness of financial incentives in changing healthcare professional behaviours and patient outcomes. Cochrane Database Syst Rev. 2011;(7):CD009255. https://doi.org/10.1002/14651858.CD009255.
5. Giuffrida A, Torgerson DJ. Should we pay the patient? Review of financial incentives to enhance patient compliance. BMJ. 1997;315(7110):703–7.
6. NHS England. NHS staff health and wellbeing: CQUIN guidnace. 2016. Retrieved 5th March 2010 from https://www.england.nhs.uk/wp-content/uploads/2016/03/HWB-CQUIN-Guidance.pdf.

7. Christianson JB, Leatherman S, Sutherland K. Review. Lessons from evaluations of purchaser pay-for-performance programs. Med Care Res Rev. 2008;65(6):5S–35S.
8. Sutherland K, Christianson JB, Leatherman SJ. Review. Impact of targeted financial incentives on personal health behavior. Med Care Res Rev. 2008;65(6):36S–78S.
9. England N. Commissioning for quality and innovation (CQUIN) Guidance for 2015/16. Leeds: Department of Health; 2015.
10. Glass C. Health Work and Well-being Challenge Fund. In: Pensions TDfWa, editor. London; 2010.
11. Black D. Working for a healthier tomorrow. In: Pensions SoSHatSoSfWa, editor. UK; 2008.
12. NHS England. NHS staff health and wellbeing: CQUIN 2017-19 Indicator 1 Implementation Support. 2018. Retrieved 5th of March 2010 from https://www.england.nhs.uk/wp-content/uploads/2018/05/staff-health-wellbeing-cquin-2017-19-implementation-support.pdf.
13. Gelman A. Prior distributions for variance parameters in hierarchical models (comment on article by Browne and Draper). Bayesian Anal. 2006;1(3):515–34.
14. Gelman A, Jakulin A, Pittau MG, Su Yu-Sung S. A weakly informative default prior distribution for logistic and other regression models. Ann Appl Stat. 2008;2(4):1360–83.
15. Li F, Lokhnygina Y, Murray DM, Heagerty PJ, DeLong ER. An evaluation of constrained randomization for the design and analysis of group-randomized trials. Stat Med. 2016;35(10):1565–79.
16. Ivers NM, Halperin IJ, Barnsley J, Grimshaw JM, Shah BR, Tu K, Upshur R, Zwarenstein M. Allocation techniques for balance at baseline in cluster randomized trials: a methodological review. Trials. 2012;13(1):120.
17. Morgan L, Rubin D. Rerandomization to improve covariate balance in randomized experiments. Massachusetts: Harvard University; 2011.
18. UK Standard Industrial Classification of Economic Activities 2007 (SIC 2007). In: Statistics OfN, editor. UK 2007.
19. Raab GM, Butcher I. Balance in cluster randomized trials. Stat Med. 2001;20(3):351–65.

Ready to submit your research? Choose BMC and benefit from:

- fast, convenient online submission
- thorough peer review by experienced researchers in your field
- rapid publication on acceptance
- support for research data, including large and complex data types
- gold Open Access which fosters wider collaboration and increased citations
- maximum visibility for your research: over 100M website views per year

At BMC, research is always in progress.

Learn more biomedcentral.com/submissions

BMC
