# Supplementary material for: Evaluation of an organisational-level monetary incentive to promote the health and wellbeing of workers in small and medium-sized enterprises: A mixed-methods cluster randomised controlled trial
Source: PLOS Glob Public Health. 2023 Jul 6;3(7):e0001381. doi: 10.1371/journal.pgph.0001381 (PMC10325111; doi:10.1371/journal.pgph.0001381)
Supplement: Attachment — Submitted filename: PGPH-D-21-00689 Authors Response .docx [file pgph.0001381.s004.docx]

**PGPH-D-21-00689
Evaluation of an organisational-level monetary incentive to promote the health and wellbeing of workers in small and medium-sized enterprises: a cluster randomised controlled trial
PLOS Global Public Health**

We would like to take this opportunity and thank you for your time and efforts in reviewing our manuscript. *Kindly note that the supplementary material numbering and word count are updated.*

Please find responses below:

**1. Please provide separate figure files in .tif or .eps format and remove any figures embedded in your manuscript file. Please also ensure that all files are under our size limit of 10MB.**

***Authors response:*** *all figures have been separately provided in a suitable format. We can confirm that there are no figures embedded in the revised manuscript.*

**2. Thank you for submitting your clinical trial and for providing the name of the registry and the registration number. The information in the registry entry suggests that your trial was registered after patient recruitment began. PLOS strongly encourages authors to register all trials before recruiting the first participant in a study**

**a) your reasons for your delay in registering this study (after enrolment of participants started);**

**b) confirmation that all related trials are registered by stating: “The authors confirm that all ongoing and related trials for this drug/intervention are registered”.**

***Authors response:*** *Kindly note that this is not a clinical trial (hence the delay in finding a suitable registry). The reasons for the delay are now provided in the revised manuscript.*

*We can confirm that there are no ongoing and related trials for this intervention. This is now stated in the revised manuscript.*

3. Please review your reference list to ensure that it is complete and correct. If you have cited papers that have been retracted, please include the rationale for doing so in the manuscript text, or remove these references and replace them with relevant current references. Any changes to the reference list should be mentioned in the rebuttal letter that accompanies your revised manuscript. If you need to cite a retracted article, indicate the article’s retracted status in the References list and also include a citation and full reference for the retraction notice.

Authors response: We can confirm that there are no updated or additional references. No change.

**4. Reviewer #1: As the study also involving qualitative technique and interviews, I don't see any data cited in the findings. It will be useful if the authors could refer to the data when explaining the qualitative data in the results section.**

**In addition, the authors wrote about thematic analysis for the qualitative data analysis. However, I don't find any example of codes that being generated by this analysis technique. Sample of codes and how it was build into themes would be better for the paper**

*Authors response: Thank you for your useful suggestions. Qualitative quotes for each of our findings are now provided in the manuscript. We also provided the coding frame in a supplementary material.*
