# Supplementary material for: Evaluation of an organisational-level monetary incentive to promote the health and wellbeing of workers in small and medium-sized enterprises: A mixed-methods cluster randomised controlled trial
Source: PLOS Glob Public Health. 2023 Jul 6;3(7):e0001381. doi: 10.1371/journal.pgph.0001381 (PMC10325111; doi:10.1371/journal.pgph.0001381)
Supplement: Attachment — Submitted filename: Reviewer_2 response_280423.docx [file pgph.0001381.s005.docx]

**Reviewer #2: Minor concern**
**Abstract**
1. Please check the title: used a mixed-methods cluster randomised trial instead of “cluster randomized trial”

*Authors response:* *Title amended.*

2. Please define reactivity effect

*Authors response: Definition inserted. In the main text we referenced the paper that defines this terminology (reference 14).*

3. The impact estimates seem not be statistically significant looking at the 95% Credible Interval. In other words, the intervention seems not to be working especially on employee health behavior or wellbeing outcomes. Please check and confirm both the point and interval estimate. Would you recommend the intervention for larger scale-up based on the impact estimate?,

*Authors response: Statistical significance is not a relevant concept for this trial both as it uses a Bayesian approach to inference (so there is no “null hypothesis”) and that we explicitly state in our protocol (doi.org/10.1186/s12889-019-6582-y) that we would not use such an approach for interpreting trial results given strong arguments against doing so (doi: 10.1080/00031305.2018.1527253). We provide both credible intervals and probabilities that the effect was positive to aid in interpretation. We note that the intervention improved the provision of services and the employees’ awareness of this provision, but did not seem to affect actual employee behaviour or wellbeing, as we note in the Results and Discussion sections of the manuscript.*

4. Check line 120, 125 and 126 for spelling errors

*Authors response: Checked and amended.*

**Major concern**
5. Authors must demonstrate in this paper how they arrived at appropriate clustering effect. What was the exact estimate of the design/clustering effect. How did the authors adjust for the multiple arm effect in the estimation of the sample size? Authors must explain how they arrive at 15 employees per SME.

*Authors response: Kindly find the full details of the sample size calculation are provided in the trial protocol ( doi.org/10.1186/s12889-019-6582-y), however we have added an additional couple of sentences in the paper to further explain the clustering and calculation. Briefly, we specified a prior for the intraclass correlation coefficient and cluster autocorrelation coefficient to average over plausible levels of clustering. We estimated the probabilities at different sample sizes using a Monte Carlo simulation-based approach.*

6.1. The study accounted for 15% attrition, but the analysis showed that 19% of the SMEs actually attritted. Authors should explain how it affected the overall results and the limitation thereof.

*Authors response: Thank you for raising this point. We discussed attrition in light of the results and limitations in the Strength and limitations section (line 629 and onwards).*

6.2. It seems the 95% credible intervals from the Bayesian models looks incredibly large and highly imprecise. It is because of the sample size?. May be a sensitivity analysis using a pure frequentist approach for the respective Hierarchical Bayesian methodology and putting the results side-by-side would be greatly beneficial in this case. This needs to be checked.

*Authors response: We would politely disagree with the reviewer that adding a new set of post-hoc analyses outside of the proposed analyses (doi.org/10.1186/s12889-019-6582-y) using a different methodology is justified. We do not think that this would address the reviewer’s concern about the size of the credible intervals. The credible intervals presented in the paper are correct.*

7. A difference in differences analysis could help address the effect of time invariant and varying covariates between the intervention and control SMEs especially when there were some covariate imbalance at baseline

*Authors response: The analysis includes indicators for baseline/endline observation, whether it had the intervention at the time, and an indicator for the “double control”. Thus, the model is already a difference-in-differences type analysis as it accounts for baseline-endline changes and intervention-control changes, and their interaction. We also include adjustment in the model for the covariates used in the restricted randomisation process, which also deals with covariate imbalance. However, in follow-up work we have also shown that covariate imbalance has little effect on precision in multi-arm cluster trials with baseline measures (doi: 10.1002/sim.8828).*

8. Please specify the empirical model used in the estimation of the model parameters

*Authors response:* *Please see section “Analysis” in the Methods section, which describes the models in detail (Bayesian generalised linear mixed models, both Binomial-logistic and linear depending on outcome) -we also provide a link to further details in the protocol that is referenced in the manuscript (doi.org/10.1186/s12889-019-6582-y).*
